# Late-Onset Urticarial and Erythema Multiforme-Like Eruption Following SARS-CoV-2 Infection

**DOI:** 10.7759/cureus.78547

**Published:** 2025-02-05

**Authors:** Barbara S Suening, Molly E Boyko, Alicia J Starr, M. Holly Glover

**Affiliations:** 1 Dermatology, University of Central Florida College of Medicine, Gainesville, USA; 2 Dermatology, Edward Via College of Osteopathic Medicine, Spartanburg, USA; 3 Internal Medicine, Inova Fairfax Medical Center, Falls Church, USA; 4 Dermatology, Grand Strand Medical Center, Myrtle Beach, USA

**Keywords:** covid-19, cutaneous urticarial eruption, erythema multiforme-like, sars-cov-2, urticarial dermatosis

## Abstract

Although SARS-CoV-2 primarily targets the upper respiratory system, its evolving clinical profile has revealed a diverse range of manifestations across multiple organ systems. Emerging evidence increasingly highlights various skin manifestations associated with the virus. This report details the case of a patient who developed a rapidly progressive urticarial dermatosis, characterized by both urticarial and erythema multiforme-like features, five weeks after a SARS-CoV-2 infection. Our objective is to underscore the histologic patterns of late-onset skin eruptions related to SARS-CoV-2, focusing on dermatological complications that can arise beyond the acute phase of the infection.

## Introduction

The novel COVID-19 resulted in a worldwide pandemic. The virus is a variant of SARS-CoV-2. According to the World Health Organization epidemiologic update in January 2024, the number of confirmed SARS-CoV-2 cases has exceeded 774 million globally [1]. The ongoing evolution of SARS-CoV-2 has revealed a range of clinical manifestations varying in degree of disease severity and numerous post-acute sequelae [2].

SARS-CoV-2 primarily impacts the respiratory system, commonly presenting with fever, body aches, loss of smell, cough, shortness of breath, runny nose, and sore throat, among other symptoms. Potential complications of the infection include, but are not limited to, acute respiratory distress syndrome, cardiac injury, and acute kidney injury [2].

Since the onset of the pandemic, there has been a significant rise in the documentation of cutaneous manifestations associated with SARS-CoV-2. However, the prevalence of cutaneous manifestations attributed to SARS-CoV-2 remains challenging to quantify. Cutaneous lesions were likely to be underreported at the start of the pandemic due to the immense outbreak, numerous critical patients, and limited research on the novel virus [3]. Various studies, including those from the early stages of the pandemic, have reported the incidence of related skin lesions ranging from less than 1% to as high as 20% [4-6]. Skin reactions attributed to SARS-CoV-2 are most often present in the acute setting of the infection, making late-onset skin reactions less common [4]. This case report discusses a late-onset dermatologic skin reaction that occurred approximately five weeks following a SARS-CoV-2 infection.

## Case presentation

A 70-year-old female presented to the dermatology clinic for evaluation of a rapidly progressive rash that was initially present on her right lower extremity. Over the course of three days, the rash sequentially spread from her lower extremities to her abdomen and then to her right elbow. She reported tenderness and warmth in the affected areas but denied any itching.

The patient’s past medical history included stage 3 chronic kidney disease, hypertension, and secondary hyperparathyroidism. Within the past three months, the patient began oral magnesium supplements and discontinued taking metolazone, spironolactone, and escitalopram. The patient reported SARS-CoV-2 infection approximately five weeks before the rash appeared and was treated with a five-day course of nirmatrelvir-ritonavir. Since the viral illness, she has experienced persistent weakness. The patient denied any recent imaging with contrast or surgeries.

**Figure 1:**
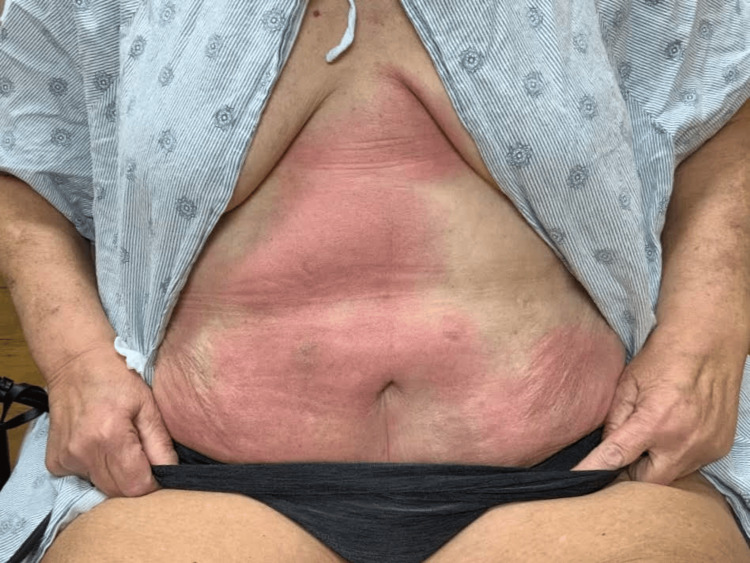
Coalescing erythematous plaques with ill-defined borders diffusely spread over the patient’s abdomen

**Figure 2:**
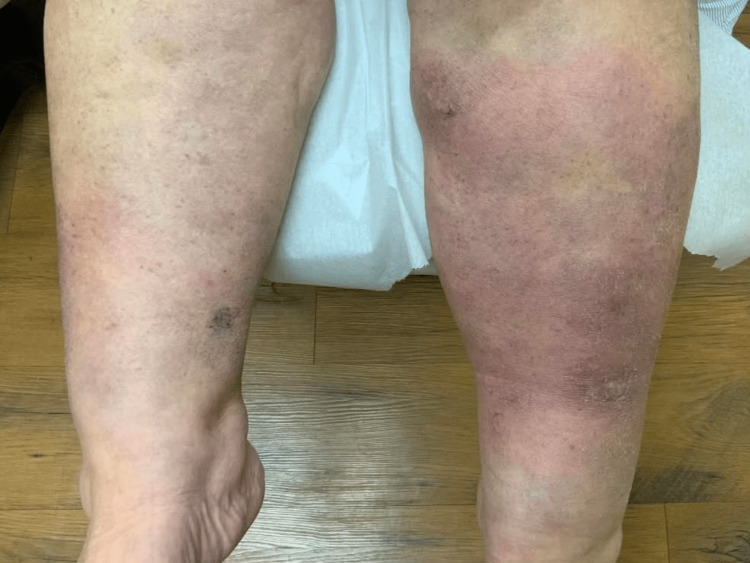
Pretibial region displaying erythematous plaques present bilaterally with more prominence on the patient’s left side

**Figure 3:**
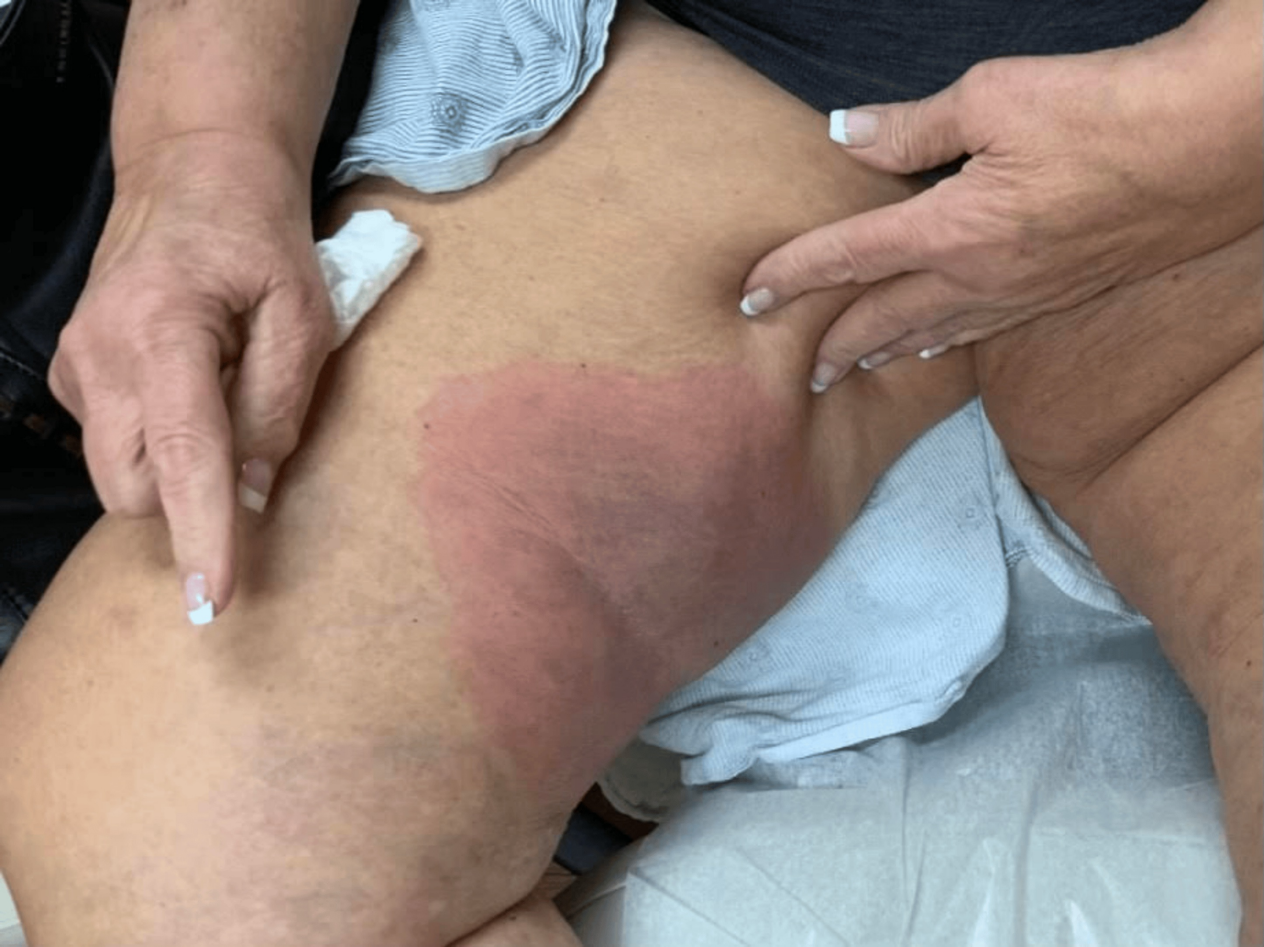
Site of the patient’s initial rash located on the right inner thigh, notable for its increased demarcation and central violaceous hue

On full-body skin examination, erythematous rashes were observed on the medial abdomen, right and left anterior tibial regions, right medial thigh, and right elbow (Figure 1, Figure 2, Figure 3). The lesions were characterized by variable-sized coalescing plaques that had diffusely spread to the areas noted. The rash on the right thigh demonstrated increased demarcation and a violaceous hue compared to the newer lesions found on the abdomen and right elbow. Overall, the rashes exhibited polymorphic features, including aspects resembling urticaria and erythema multiforme. Aside from the skin examination, the remainder of the patient’s physical exam was largely unremarkable, with no significant findings in other systems.

Further workup included an evaluation of recent lab work from four weeks prior, which showed an elevated parathyroid hormone intact of 135 pg/mL and ionized calcium of 4.9 mg/dL. The basic metabolic panel showed a urea nitrogen level of 30 mg/dL, a creatinine level of 1.43 mg/dL, an estimated glomerular filtration rate of 36 mL/min/1.73 m², and magnesium of 1.9 mg/dL. Complete blood count showed a white blood cell count of 6.5 K/uL, hemoglobin of 12.2 g/dL, and platelets of 317 K/uL. The thyroid panel was within normal limits.

A 4 mm punch biopsy of the patient’s left mid-abdomen, performed at the outpatient clinic, revealed perivascular and interstitial dermatitis with mixed features. The biopsy revealed a nonspecific cutaneous reaction pattern, noting superficial and deep perivascular and interstitial mixed infiltrates consisting of lymphocytes, histiocytes, neutrophils, and occasional eosinophils. Superficially, focal intralymphatic histiocytic proliferation was evident. On histologic conventional sections, the cells appeared as intravascular lymphoid cells with prominent cytoplasm, raising suspicion for an intravascular lymphoproliferative process. A panel of immunohistochemical stains, including markers for lymphoid and histiocytic cells, was conducted to exclude intravascular lymphoproliferative disorder. The results confirmed a reactive intralymphatic histiocytosis pattern, indicating an inflammatory response rather than a malignant condition.

The overlapping histopathologic patterns observed included urticarial, granulomatous, lymphocytic vasculitis, and intralymphatic histiocytosis. Based on the clinical context and histological findings, the rash was diagnosed as a late SARS-CoV-2-associated urticarial reaction. Other infectious, autoimmune, and allergic conditions were systematically excluded based on the patient’s clinical presentation and history. The patient had been previously healthy and developed the rash following SARS-CoV-2 infection, suggesting a viral rather than an autoimmune or allergic etiology. There were no signs of recurrent symptoms or complications typically associated with autoimmune disorders, and in the absence of concerning symptoms or a prior history of allergies, additional testing for these conditions was deemed unnecessary. The patient’s SARS-CoV-2 vaccination status was not documented at the time of evaluation, and follow-up beyond the initial visit was limited. However, at the last documented encounter, the patient reported a complete resolution of symptoms approximately one week after initiating oral prednisone therapy.

## Discussion

Nonspecific findings with overlapping histopathologic patterns, including urticarial, granulomatous, lymphocytic vasculitis, and intralymphatic histiocytosis, have been previously described in polymorphic late SARS-CoV-2 infection-associated urticarial eruptions [3,7]. Common viral infections were also associated with nonspecific inflammatory-type rashes, which may present in various patterns, such as confluent erythematous, urticaria, morbilliform, and maculopapular [3].

A retrospective study from Spain utilized a nationwide collection of case images and clinical data to identify the most common dermatologic patterns associated with SARS-CoV-2. These patterns, listed in order of decreasing prevalence, included erythema with pustules or vesicles, urticaria, maculopapular eruptions, and livedo or necrosis [8]. The study also explored the temporal aspects of cutaneous findings, revealing that urticarial and maculopapular lesions appeared similarly, with approximately 5% of patients presenting cutaneous signs before other clinical manifestations, 61% simultaneously, and about 34% afterward [8]. One limitation of the study is the variability in the onset of skin lesions following the initial SARS-CoV-2 infection, as well as the lack of specificity regarding this time frame. The delayed recognition of skin manifestations, often occurring a week or more after the initial infection, may result from limited awareness of their association with SARS-CoV-2, given that such occurrences are not widely documented.

A case series involving three elderly patients with late-onset SARS-CoV-2 cutaneous manifestations demonstrated a histological pattern consistent with the findings in this report. The rashes, described as erythematous with urticarial and erythema multiforme-like features, appeared 28 to 38 days after the initial positive SARS-CoV-2 test. Histological examinations revealed a dermatitis pattern characterized by perivascular lymphocytic infiltrate, with histiocytes present in both the epidermis and perivascular areas [4]. Additionally, another case series noted a common perivascular infiltrate pattern in four patient biopsies, with maculopapular eruptions occurring 20 to 38 days post-infection [9]. It is essential to recognize that lymphocytic vasculitis, a histopathologic pattern often associated with viral infections, differs from other types of vasculitis, such as leukocytoclastic or nodular vasculitis, in its clinical implications [10,11]. However, the precise mechanisms and histopathology underlying these cutaneous findings remain unclear.

Cutaneous eruptions associated with viral infections are often attributed to the infection itself or its residual antigens [9]. In the case of SARS-CoV-2, it has been hypothesized that late-onset urticarial and erythema multiforme-like rashes may arise from a delayed immune response to the virus, characterized by a type IV hypersensitivity mechanism, rather than from direct viral effect [4,9]. Initially, the viral infection stimulates the immune system, activating T cells and producing antibodies. Following recovery, some individuals retain immune memory against viral antigens. In certain cases, this immune memory can lead to a misdirected response, triggered by remnants of the virus or residual antigens. As a result, activated T cells release inflammatory mediators, including histamine from mast cells, which manifests as an urticarial rash. This delayed reaction reflects the complex interplay of the immune system in the aftermath of viral infections.

The role of angiotensin-converting enzyme 2 (ACE2), a key receptor for SARS-CoV-2, is also under investigation. Some theories suggest that skin cells expressing ACE2 may influence cutaneous manifestations. The ACE2 receptor, known to be the entry point for the SARS-CoV-2 virus, may impact how the skin responds to certain conditions or symptoms related to the virus. In the context of the skin, ACE2 expression can affect various processes, including inflammation, immune response, and vascular functions. Thus, when skin cells interact with the virus or are influenced by it, they may contribute to specific skin symptoms, highlighting ACE2’s role not only in the respiratory system but also in the skin’s response to pathogens [12,13].

Additionally, atypical drug reactions, whether to therapeutic agents or a combination of drugs and viral interactions, have been explored [9]. In this instance, although a drug reaction was initially considered due to recent changes in medication, the resolution of symptoms without reintroducing prior medications or discontinuing oral magnesium suggests this is unlikely. Given the relative novelty of this field, current findings from existing studies remain limited, and further research is warranted to clarify these mechanisms.

Topical steroids are frequently prescribed for various inflammatory rashes, and reports have noted symptom relief in patients with skin lesions following SARS-CoV-2 infection [14]. Other treatment options for SARS-CoV-2-related skin lesions include oral antihistamines and low-dose corticosteroids [15]. A case series reported that a patient who received 30 mg of prednisone with a weekly tapering dose of 10 mg recovered after a 10-day treatment course [16]. The improved symptom management following corticosteroid treatment can be considered when exploring therapeutic options.

## Conclusions

While cutaneous manifestations of SARS-CoV-2 in the acute phase are well documented, delayed reactions are less studied. This case report describes a rare late-onset dermatological manifestation of SARS-CoV-2: an urticarial dermatosis with erythema multiforme-like features occurring five weeks post-infection. Histological findings showed a nonspecific pattern with characteristics of urticaria, granulomatous dermatitis, and lymphocytic vasculitis. The potential connection between SARS-CoV-2, delayed immune responses, and ACE2 receptors in these skin manifestations warrants further investigation. Although drug reactions were considered, the resolution of symptoms without reintroducing previous medications or discontinuing oral magnesium makes this less likely.

This case highlights the importance of documenting SARS-CoV-2-related skin manifestations to aid clinicians in diagnosing idiopathic skin eruptions. It underscores the need for further research to understand late-onset skin reactions and to broaden our knowledge of SARS-CoV-2’s dermatological effects. Future studies should aim to clarify the relationship between SARS-CoV-2 and delayed skin reactions, refine diagnostic criteria, and develop effective treatment strategies.
